# Genomic analysis reveals low genetic diversity and no continuous reintroduction of piscine myocarditis virus in farmed Atlantic salmon in the Faroe Islands

**DOI:** 10.1099/jgv.0.002068

**Published:** 2025-02-12

**Authors:** Maria Marjunardóttir Dahl, Petra Elisabeth Petersen, Debes Hammershaimb Christiansen

**Affiliations:** 1 Faroese Food and Veterinary Authority, National Reference Laboratory for Fish and Animal Diseases, V.U. Hammershaimbsgøta 11, Tórshavn, Faroe Islands

**Keywords:** Atlantic salmon, cardiomyopathy syndrome, genetic diversity, next-generation sequencing (NGS), piscine myocarditis virus, Totiviridae, transmission selection, whole-genome sequencing

## Abstract

Piscine myocarditis virus (PMCV) is the causative agent of cardiomyopathy syndrome (CMS), a significant disease in farmed Atlantic salmon (*Salmo salar* L.). Although an increasing number of CMS outbreaks have been recorded in the Faroe Islands since the reemergence of CMS in 2013, overall PMCV genetic diversity, transmission pathways and evolutionary trajectories remain elusive. Here, we present a fast amplicon-based whole-genome sequencing method of PMCV directly from field samples and disclose 48 novel genomes, adding to the single genome currently available. Phylogenetic analysis revealed that genomes with a broad spatiotemporal representation of Faroese farmed salmon formed a homogenous monophyletic cluster compared to Norwegian and Irish PMCV genomes. Homogeneity of the Faroese genomes was substantiated with principal component analyses, where no spatiotemporal clustering of genotypes was found, nor any clustering based on roe or smolt origin. One genome from a returning wild salmon differed considerably from all the rest and formed an outgroup. All three ORFs exhibited signs of purifying selection, although ORF3 displayed a comparatively lower degree of selective constraint. Furthermore, no virulence-determining amino acid substitutions were identified in the Faroese genomes as no association was found between CMS cases and specific amino acid substitutions or motifs. Our data suggest that PMCV was introduced into the Faroe Islands from Norway, where brood fish is known to be infected. However, despite a steadily increasing import of Norwegian roe, our results show no continuous reintroduction of persisting PMCV strains to Faroese farmed salmon.

## Data Summary

The authors confirm that all supporting data and protocols have been provided within the article or through supplementary data files. The PMCV genomes have been deposited in the GenBank® database under accession numbers PP525955–PP526002.

## Introduction

Cardiomyopathy syndrome (CMS) is a serious cardiac disease in farmed Atlantic salmon, characterized by severe and chronic progressive cardiac myodegeneration. In 2009, it was demonstrated that CMS is a transmittable disease [1,2], and in 2010, the piscine myocarditis virus (PMCV) was identified as the causative agent [3,4]. The first reports of CMS were made in Norway in 1985 [5] and subsequently in the Faroe Islands in 1992 [6], Scotland in 1997 [7] and Ireland in 2012 [8]. Cases of CMS were reported in the Faroe Islands all through the 90s, but after a near shutdown of the industry in the early twenty-first century, caused by massive outbreaks of infectious salmon anaemia (ISA), there were no reports of CMS and no PMCV detection until 2013 [9], despite extensive surveillance [10,11; Faroese Food and Veterinary Authority (FFVA), unpublished data]. CMS or PMCV has not been detected in Chile [12] or Iceland [13].

CMS usually develops slowly over several months prior to the clinical phase, during which severe degeneration of the heart muscle, particularly in the atrium and ventricle, becomes apparent [14]. The disease has mainly caused mortality in large Atlantic salmon in the second year at sea resulting in substantial economic losses for the industry [9,15]. However, recent reports have described the disease shortly after sea transfer [16]. CMS has, for many years, been one of the most important diseases in Norwegian aquaculture, and cases are increasing in Scotland and Ireland [17,18]. Similarly, the number of CMS cases has increased markedly within the last decade in the Faroe Islands (FFVA, unpublished data).

PMCV is a double-stranded RNA virus resembling viruses of the Totiviridae family that infect protozoan parasites and fungi [19]. The non-segmented genome of 6.7 kb encompasses three ORFs [3]. ORF1 encodes a protein of 861 aa suggested to represent the capsid protein, and ORF2 encodes a protein of 726 aa believed to be the RNA-dependent RNA polymerase. Pending on a slippery site in the 3′ end of ORF1, frameshifting may also cause ORF2 to be translated as a fusion protein with ORF1 [3,20]. ORF3 encodes a protein of 302 aa which shares no sequence homology with proteins of known totiviruses and whose function is yet unknown [3,19], though it is hypothesized to play a role in the ability to infect more advanced hosts compared to the true totiviruses [20]. Wiik-Nielsen *et al.* [21] suggested amino acid positions 84, 87 and 97, containing either Isoleucine-Lycine-Arginine (IKR) or Valine-Glutamine-Glutamine (VQQ), as a putative virulence motif.

Little is known about the natural reservoirs of PMCV. Although PMCV has been detected in a few wild Atlantic salmon, this reservoir seems to be of minor importance [19].

There is currently no treatment for CMS and no effective cultivation system for PMCV, impeding vaccine development. Quantitative trait locus (QTL)-selected roe with CMS resistance has been on the market for a decade, and there is an increased focus on cardiovascular health since deviating heart morphology can be associated with the risk of CMS-related cardiac rupture [19,22]. Additional risk factors for developing CMS include sea transfer in late fall, origin of smolt, time at sea and previous outbreaks of pancreas disease (PD) or heart and skeletal muscle inflammation [23]. Lastly, general stress from mechanical delousing, such as crowding and pumping, is also widely seen as a risk factor for developing CMS [17]. The number of delousing events in the Faroe Islands has steeply increased over the past decade, with a near-complete shift from chemical to mechanical treatments by 2018 [24].

Faroese farming companies receive fertilized roe from Iceland and Norway; however, whereas PMCV has never been detected in Iceland [13], PMCV-infected brood fish has been a widespread and persistent problem in Norway for many years [17]. Garseth *et al.* [19] suggested that the reemergence of PMCV in the Faroes in 2013 could be explained by roe imported from Norway. Previous genetic studies have not considered the roe and smolt origin of samples and solely relied on analysis of ORF1 and/or ORF3. These studies have found PMCV to be genetically homogenous in both Norway [21] and Ireland [25].

Infectious diseases are a big threat to the growing aquaculture industry, and whole-genome sequencing approaches can provide an in-depth understanding of the epidemiology and mitigation of these diseases [26,27]. With only one publicly available PMCV genome [3], we still lack the tools to thoroughly understand CMS disease development, including transmission mode and the potential evolution of PMCV virulence. The aim of this study was to provide a comprehensive genetic characterization of the PMCV virus in the Faroe Islands, tracking its reemergence in 2013 and subsequent increase and spread. We developed a fast amplicon-based whole-genome sequencing method for field samples, generated 48 high-quality PMCV genome sequences and conducted a phylogenetic analysis that revealed a distinct Faroese PMCV clade. To contextualize the phylogenetic data, we collected information on roe and smolt origin, sampling site and date, cycle threshold (Ct) values and CMS status for all samples in this study (collectively referred to as metadata).

## Methods

### Production overview

The Faroese salmon farming industry is organized into 28 marine farming sites (managemental zones with a minimum separation distance of 5 km), 8 freshwater smolt farms using freshwater only and 2 brood fish production sites, owned by three companies. Production of smolt and brood fish is fully land-based in indoor facilities. The industry was until 2005 supplied with roe exclusively from Faroese brood fish producer FO1. From 2005 and onwards, the farms were supplied with roe from Faroese brood fish (FO1 between 1990 and 2016 and FO2 from 2017 onwards) as well as increasing amounts of imported roe from Icelandic (ICE) and Norwegian (NO1, NO2 and NO3) producers. For this study, data on imported roe were presented from 2008 onwards to include a sufficient time period before the reemergence of CMS in the Faroes in 2013 (Table S1 available in the online Supplementary Material). Whereas PMCV has never been detected in FO1 and ICE brood fish, it is consistently detected in FO2 as well as Norwegian brood fish (FFVA, unpublished data [17]. All three companies have imported roe from Iceland and one or more Norwegian producers. None of the smolt farms have exclusively sourced roe from a single producer in this study period.

### Sample selection

**Fig. 1.**
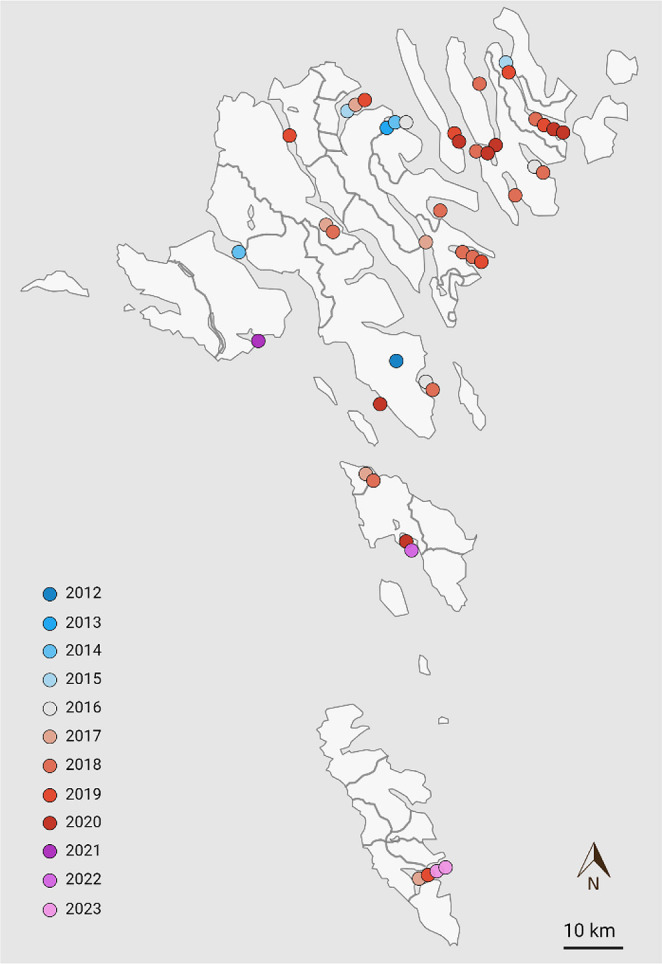
Map of the Faroe Islands showing sampling sites of the Faroese samples included in this study. Each dot represents one sampling, and the colouring indicates the sampling year as seen in the bottom-left corner.

This study is based on samples originating from 22 salmon farming production sites as well as a returning wild Atlantic salmon, all collected by the authorities or the farming companies over a period of 12 years (Fig. 1, Table 1).

**Table 1.** PMCV samples sequenced for this study by country of origin and in chronological order from the first detection of PMCV in 2012. Sample names are as follows: prefixes denote country of origin; FO for the Faroe Islands and NO for Norway. Numbers between slashes are generated by an internal registration system for anonymity, and finally, suffixes are sampling year

Sample name	CMS	Accession no.	Country	Location	Sampling date	Sample type	Ct
FO/1014-18/2012		PP525955	Faroe Islands	Wild fish I	December 2012	Heart, head kidney, gill, spleen and pancreas	17
FO/819-19/2013		PP525956	Faroe Islands	Marine farm XII	October 2013	Heart, head kidney and gill	25
FO/498-19/2014	†	PP525957	Faroe Islands	Marine farm XII	May 2014	Heart, head kidney and gill	15
FO/847-02/2014	†	PP525958	Faroe Islands	Marine farm XVII	August 2014	Heart and head kidney	16
FO/1414-05/2015	†	PP525959	Faroe Islands	Marine farm XIV	September 2015	Heart‡	20
FO/1418-07/2015		PP525960	Faroe Islands	Marine farm XVI	September 2015	Heart‡	25
FO/1164-01/2016		PP525961	Faroe Islands	Marine farm II	July 2016	Heart and head kidney	24
FO/1245-02/2016	†	PP525962	Faroe Islands	Marine farm XII	August 2016	Heart, head kidney and gill	20
FO/1640-02/2016		PP525963	Faroe Islands	Marine farm XIII	October 2016	Heart	18
FO/820-06/2017	†	PP525964	Faroe Islands	Marine farm XIV	June 2017	Heart	20
FO/889-13/2017	†	PP525965	Faroe Islands	Marine farm XI	July 2017	Heart	22
FO/1550-01/2017	†	PP525966	Faroe Islands	Brood fish farm II	October 2017	Heart, head kidney and gill	17
FO/1581-04/2017	†	PP525967	Faroe Islands	Marine farm XVII	October 2017	Heart, head kidney and gill	18
FO/1725-47/2017		PP525968	Faroe Islands	Brood fish farm I	November 2017	Roe fluid	28
FO/104-06/2018	†	PP525969	Faroe Islands	Marine farm IV	February 2018	Heart	16
FO/143-02/2018		PP525970	Faroe Islands	Marine farm I	February 2018	Heart	22
FO/169-33/2018		PP525971	Faroe Islands	Marine farm I	February 2018	Heart	27
FO/252-01/2018	†	PP525972	Faroe Islands	Marine farm XV	March 2018	Heart	15
FO/416-05/2018		PP525973	Faroe Islands	Marine farm V	April 2018	Swab along the lateral line	26
FO/783-04/2018	†	PP525974	Faroe Islands	Brood fish farm II	August 2018	Heart, head kidney and gill	18
FO/1085-01/2018	†	PP525975	Faroe Islands	Brood fish farm I	October 2018	Heart, head kidney and gill	16
FO/1137-24/2018	†	PP525976	Faroe Islands	Marine farm II	October 2018	Heart, head kidney and gill	15
FO/1235-21/2018	†	PP525977	Faroe Islands	Marine farm VIII	November 2018	Heart, head kidney and gill	15
FO/1243-26/2018	†	PP525978	Faroe Islands	Marine farm VII	November 2018	Heart, head kidney and gill	20
FO/1412-25/2018	†	PP525979	Faroe Islands	Marine farm XIII	December 2018	Heart, head kidney and gill	17
FO/214-01/2019	†	PP525980	Faroe Islands	Marine farm III	February 2019	Heart	16
FO/540-07/2019	†	PP525981	Faroe Islands	Marine farm XVI	June 2019	Heart, head kidney and gill	17
FO/601-04/2019	†	PP525982	Faroe Islands	Marine farm I	June 2019	Heart	17
FO/607-25/2019		PP525983	Faroe Islands	Smolt farm I	June 2019	Kidney‡	29
FO/609-02/2019		PP525984	Faroe Islands	Marine farm XIV	June 2019	Heart, head kidney and gill	25
FO/616-08/2019		PP525985	Faroe Islands	Marine farm VI	June 2019	Heart, head kidney and gill	22
FO/646-01/2019	†	PP525986	Faroe Islands	Marine farm X	July 2019	Heart and head kidney	18
FO/187-04/2020		PP525987	Faroe Islands	Marine farm IV	February 2020	Seawater§	23
FO/187-13/2020	†	PP525988	Faroe Islands	Marine farm IV	February 2020	Seawater§	23
FO/275-07/2020		PP525989	Faroe Islands	Smolt farm I	March 2020	Heart, head kidney and gill	18
FO/651-03/2020		PP525990	Faroe Islands	Marine farm XVIII	June 2020	Heart, head kidney and gill	22
FO/760-01/2020	†	PP525991	Faroe Islands	Marine farm XIX	August 2020	Heart and head kidney	18
FO/782-06/2020	†	PP525992	Faroe Islands	Marine farm VII	September 2020	Heart, head kidney and gill	16
FO/782-15/2020	†	PP525993	Faroe Islands	Marine farm VII	September 2020	Heart, head kidney and gill	21
FO/156-06/2021		PP525994	Faroe Islands	Marine farm IX	March 2021	Head kidney	24
FO/511-08/2022		PP525995	Faroe Islands	Marine farm XIX	August 2022	Heart, head kidney and gill	25
FO/01-02/2023		PP525996	Faroe Islands	Marine farm XVII	December 2022	Heart, head kidney and gill	19
FO/40-02/2023		PP525997	Faroe Islands	Marine farm XVII	January 2023	Heart and head kidney	19
NO/2107-03/2019	†	PP525998	Norway	Vestlandet	2017	Heart and head kidney	26
NO/661-02/2020	†	PP525999	Norway	Infection challenge*	July 2020	Heart	23
NO/684-17/2020	†	PP526000	Norway	Infection challenge*	July 2020	Heart	16
NO/003-07/2021	†	PP526001	Norway	Møre/Romsdal	2018	Heart and head kidney	19
NO/004-04/2022	†	PP526002	Norway	Møre/Romsdal	2018	Heart and head kidney	20

originates from a CMS outbreak in Vestlandet, Norway in 2015 and has undergone several infection challenges since.*Originated from a CMS outbreak in Vestland, Norway, in 2015 and has undergone several infection challenges since.

Pool of five fish.†CMS, as confirmed by gross pathology.

Pool of 10 fish.‡Pool of five fish.

CMS, as confirmed by gross pathology.§Pool of ten fish.

They represent all three farming companies in the Faroe Islands and are evenly distributed among the companies based on total PMCV prevalence on their respective sites. Samples are from routine inspection as well as from clinical outbreaks (Table 1). Between 2013 and 2018, all PMCV-positive production sites with samples containing sufficient PMCV viral loads for genome sequencing were included to characterize the reemergence and subsequent spread of PMCV. Between 2019 and 2023, only a subset of sites was included. The wild salmon sample was included to investigate any potential role of wild salmon as vector species. Forty-seven wild Atlantic salmon were captured alive for stripping in a restocking river, and only one tested PMCV positive with sufficient viral load for genome sequencing. For comparative purposes, the study also comprised three Norwegian samples from field outbreaks and two from an infection challenge (originating from a field outbreak), kindly provided by the Norwegian Veterinary Institute and VESO Norway, respectively (Table 1). Sample types were tissue, roe, two seawater samples and a single swab along the lateral line. Three of the tissue samples comprised a pool of five fish (Table 1). The seawater samples were part of an in-house validation of using environmental RNA/DNA (eRNA/eDNA) as a non-lethal sampling strategy for monitoring PMCV and other potential pathogens at the pen level. The samples were collected during mandatory sea louse (*Lepeophtheirus salmonis*) counts, where ten salmon from each net pen were taken into a tank with seawater and tricaine methanesulfonate for anaesthetization. One litre of the respective tank water, representing ten individuals, was sampled from two net pens – one pen with subclinical CMS but confirmed PMCV infection and one pen with clinical CMS as confirmed by gross pathology (Table 1). The water samples were filtrated on the day of collection by membrane filtering through a 0.45 µm pore size filter on a WaterVac 100 (Rocker Scientific) and submerged in RNA Lysis buffer (RLT) lysis buffer for RNA extraction [see the RNA extraction and quantitative reverse transcription PCR reaction section].

### Reference genomes

Seventeen reference genomes were used in the phylogenetic analysis: 14 genomes from seven CMS cases in Norwegian salmon farms (A–H) [28] and 3 genomes, F/42/17, F/45/18 and F/46/18, from previously described Irish CMS outbreaks [25], kindly provided by the Marine Institute in Galway, Ireland. Accession numbers for Norwegian reference genomes can be found in Table S2.

### RNA extraction and quantitative reverse transcription PCR

All samples were collected and homogenized in RLT lysis buffer, and RNA was extracted as previously described [10,11]. Samples were screened for PMCV by a duplex one-step real-time reverse transcription quantitative PCR (RT-qPCR) to a duplex one-step quantitative reverse transcription PCR (RT-qPCR) assay that amplified and detected a PMCV-specific fragment as well as a specific fragment of the Atlantic salmon reference gene elongation factor (EF1α). The PMCV duplex RT-qPCR assay was like the Infectious Salmon Anaemia Virus (ISAV) assay described by [10,11, 29] but used PMCV-specific primers and probes [3] instead of ISAV-specific ones. The thermal cycling conditions on QuantStudio 5 (Thermo Fisher) were as outlined by Christiansen *et al.* [29].

### Primer design and multiplex PCR

Primers for multiplex amplification of PMCV genomes were designed using the online tool Primal Scheme, which generates primer pairs optimized for multiplexing by accounting for factors such as GC content, estimated secondary structures and primer-dimer formations [27]. The initial primer set was designed in 2017 with the only available whole genome from Norway, AL V-708 [3] as primary template and partial sequences from samples obtained in the Faroes as subsequent references to incorporate as much Faroese genetic diversity as possible. Fragment length and overlap parameters in Primal Scheme were set to 500 bp and 75 bp, respectively. This resulted in 18 primer pairs with fragment lengths and overlaps varying from 455 to 536 bp and 75 to 159 bp, respectively, covering 98.5% of the genome (primer set I). The Faroese PMCV genomes sequenced with primer set I exhibited significant divergence from AL V-708. To better target the Faroese genomes, we designed a second primer set (primer set II) based solely on Faroese genome sequences (primer set II). This resulted in 18 different primer pairs with fragment lengths and overlaps varying from 472 to 548 bp and 75 to 162 bp, respectively, covering 99.4% of the genome. Common for both primer sets was that primer pairs were divided into two primer pools, pool 1 and pool 2, with overlapping primer pairs in different pools. Primer sets used for specific samples can be found in Table S3.

Following a denaturation step of 65 °C for 5 min, first strand synthesis was performed on total RNA with either Superscript III (Invitrogen) or LunaScript (New England Biolabs) according to the manufacturer’s recommendations. Two multiplex PCRs with primer pools 1 and 2 were set up for each sample. Reactions contained 2.5 µl of cDNA, 12.5 µl of Q5 High-Fidelity 2X Master Mix (New England Biolabs), primer pool (1 or 2) at a final concentration of 400 nM (22.2 nM for individual primers) and added molecular grade water to a final volume of 25 µl. Temperature profiles were 98 °C for 30 s followed by 40 cycles of 98 °C for 15 s and 65 °C for 5 min.

### Next-generation sequencing on Illumina and Oxford Nanopore platforms

All quantification was made on the Qubit 4 Fluorometer (Invitrogen), and all clean-up steps were performed with AMPure beads (Beckman Coulter). For Illumina sequencing, an initial 0.8 × post-PCR clean-up was followed by library preparation performed with the KAPA Hyper library preparation kit (Roche) according to the manufacturer’s protocol using SureSelect^XT2^ indexing adapters (Agilent). Completed libraries were sequenced with the MiSeq Reagent Kit v3 on a MiSeq (Illumina). For Nanopore sequencing, an initial 1.0× post-PCR clean-up was followed by library preparation using either Nanopore I: the Ligation Sequencing Kit (Oxford Nanopore), NEBNext Companion Module for Ligation Sequencing (New England Biolabs) and the Native Barcoding Expansion 1–12 and 13–24 kits (Oxford Nanopore) following the protocol (beginning with the end-prep step) outlined in ‘PCR tiling of SARS-CoV-2 virus’ (version: PTC_9096_v109_revL_06Feb2020; Oxford Nanopore) or Nanopore II: Native Barcoding kit 24 V14 (Oxford Nanopore) according to manufacturer’s protocol. Completed libraries were loaded onto R9.4.1 (Nanopore I) or R10.4.0 (Nanopore II) flow cells and sequenced on a GridION (Oxford Nanopore). Protocols for specific samples can be found in Table S3.

### Bioinformatics

Illumina data basecalling, demultiplexing, adapter trimming and quality assessment were made on the MiSeq. Illumina reads were imported as paired reads into the CLC Genomics Workbench (v12.0.3) (Qiagen). Nanopore data were basecalled with Guppy on the GridION and imported into the CLC Genomics Workbench (v22.0.2). Imported reads from both platforms were mapped to the reference genome, AL V-708 [3] with default parameters. Consensus sequences were extracted using a quality score with a noise threshold of 0.1. Additional basecalling was made manually with the help of mapping files. Multiple sequence alignment was performed with the parameters, gap open cost=10, gap extension cost=1 and end gap cost=cheap. A maximum likelihood tree was generated based on the best fitting substitution model, General Time Reversible with rate and topology variation, suggested by CLC Genomics Workbench (v22.0.2) with bootstrap values calculated from 1000 replicates. A pairwise comparison was performed on CLC Main Workbench (v.24.0).

### Statistical analysis

A number of mapped-to-total reads were fitted by simple linear regression and back-extrapolated to zero reads. Correlation between the two was assessed by a two-tailed Pearson correlation test using GraphPad Prism version 9.5.0. The selection pressure (*ω*) was estimated for the coding sequences (ORFs) by calculating the ratio between non-synonymous (dN) to synonymous mutations (dS). Values >1 indicate positive selection, =1 indicate neutral evolution and <1 indicate purifying selection; *ω*=dN/dS with Single Likelihood Ancestor Counting (SLAC) in Datamonkey [30]. *Z*-tests were performed to test for neutral selection (null hypothesis), positive selection and purifying selection. The tests, averaging over all sequence pairs, were conducted in mega11 [31] using the Kumar method [32], with the variance of differences calculated using 1000 bootstrap replicates.

Principal component analysis (PCA) on informative SNP loci (rare allele coded 1 and common allele 0) [33] was applied as an independent means to visualize potential clustering of genotypes in relation to roe and smolt origin, sampling site and year. PCA was performed on the covariance matrix, and principal components (PC) were selected from parallel analysis. Biplots of PC1 and PC2 were constructed. PCA plots were created in GraphPad Prism version 9.5.0.

## Results

### High-quality amplicon-based genome sequencing of PMCV generated on Illumina MiSeq and Oxford Nanopore Gridion platforms

**Fig. 2.**
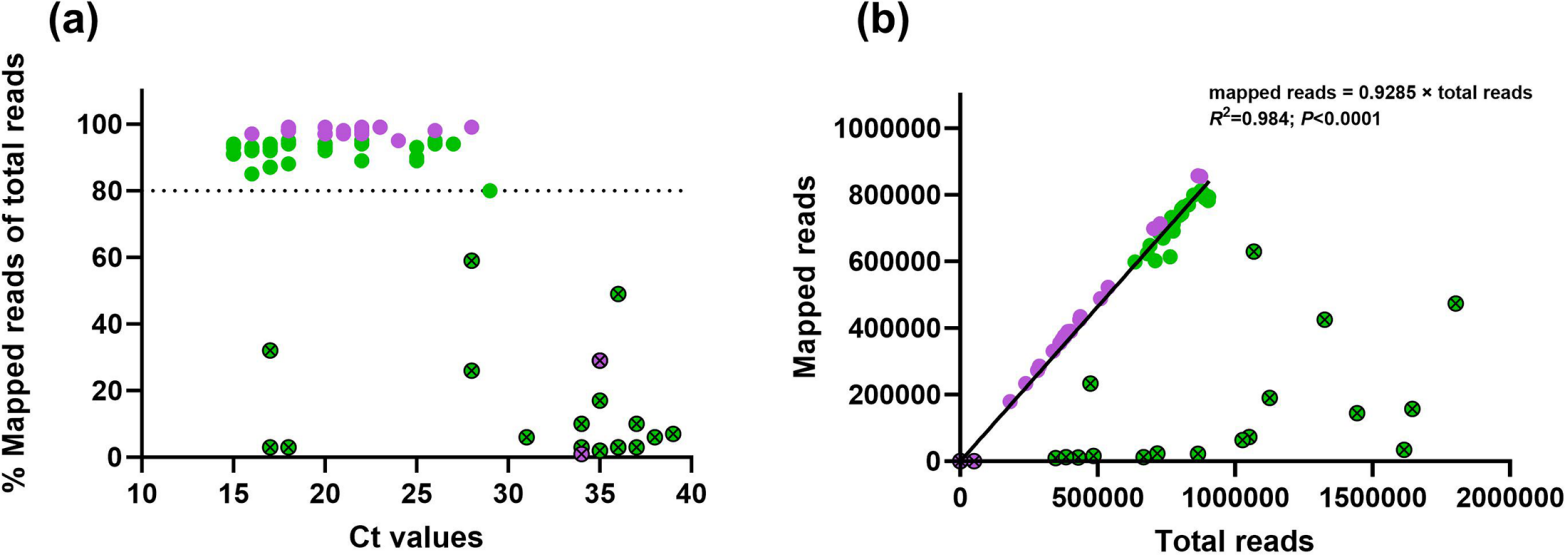
Presentation of all sequencing data retrieved from Illumina (green dots) and Nanopore (purple dots). ⨂ indicates samples that were excluded in this study. (**a**) The percentage of mapped reads to total reads was calculated for samples and set to Ct values. The threshold for valid data was set at >80% mapped reads and was not seen in Ct value >29. (**b**) Taken only the validated data points from 2a, a linear relationship was observed between the total number of reads and the number of mapped reads per sample with the following equation: mapped reads=0.9285×total reads (*R*^2^=0.984; *P*<0.0001). Two data points with total reads above 3.5×10^6^ were excluded, as they collapsed the graph.

Initially, a total of 69 samples with PMCV Ct values ranging from 15 to 39 were subjected to amplicon-based whole-genome sequencing on the Illumina MiSeq (45 samples) or the Oxford Nanopore GridION (24 samples). Because of the large amount of data generated by a next-generation sequencing (NGS) run, full genome sequences were achieved with all samples, but duplicates of samples with high Ct values (>34) revealed inconsistencies that suggested contamination and/or barcode bleeding from other samples in the same run on both platforms (data not shown). To set a threshold for valid data, the percentage of mapped reads to total reads was calculated and compared to Ct values (Fig. 2a).

Data from all samples with Ct values >29 and five with Ct values <29 had <80% mapped reads (Fig. 2a). Of the five samples with Ct values <29, three were sequenced again yielding >80% mapped reads and generated high-quality consensus sequences compared to their first-run counterparts. When excluding all samples with <80% mapped reads, 48 high-quality PMCV genomes remained where a linear relationship was observed between the total number of reads and number of mapped reads per sample (*R*^2^=0.984; *P*<0.0001) (Fig. 2b). Results from previous Sanger sequencing of ORF1 and ORF3 from a subset of samples revealed 100% identity with their corresponding NGS sequences that adhere to abovementioned criteria (highest compared Ct value of 29 – data not shown). Sequencing and mapping yields for all included samples can be found in Table S3.

### Phylogenetic analyses revealed a highly homogenous monophyletic cluster – the Faroese clade

The 48 high-quality PMCV genomes (accession numbers PP525955–PP526002, Table 1) sequenced in the present study included 42 from Faroese farmed salmon, 3 from Norwegian CMS outbreaks, 2 from a Norwegian infection challenge and 1 from a Faroese returning wild Atlantic salmon (Table 1). The 42 genomes from Faroese farmed salmon represent a broad spatiotemporal sample collection over 11 years including samples from two brood fish production sites, one out of eight smolt farms and 19 out of 28 marine farming sites. They represent about a third of all PMCV-positive production sites for each of the three production companies (Fig. 1, Table 1).

**Fig. 3.**
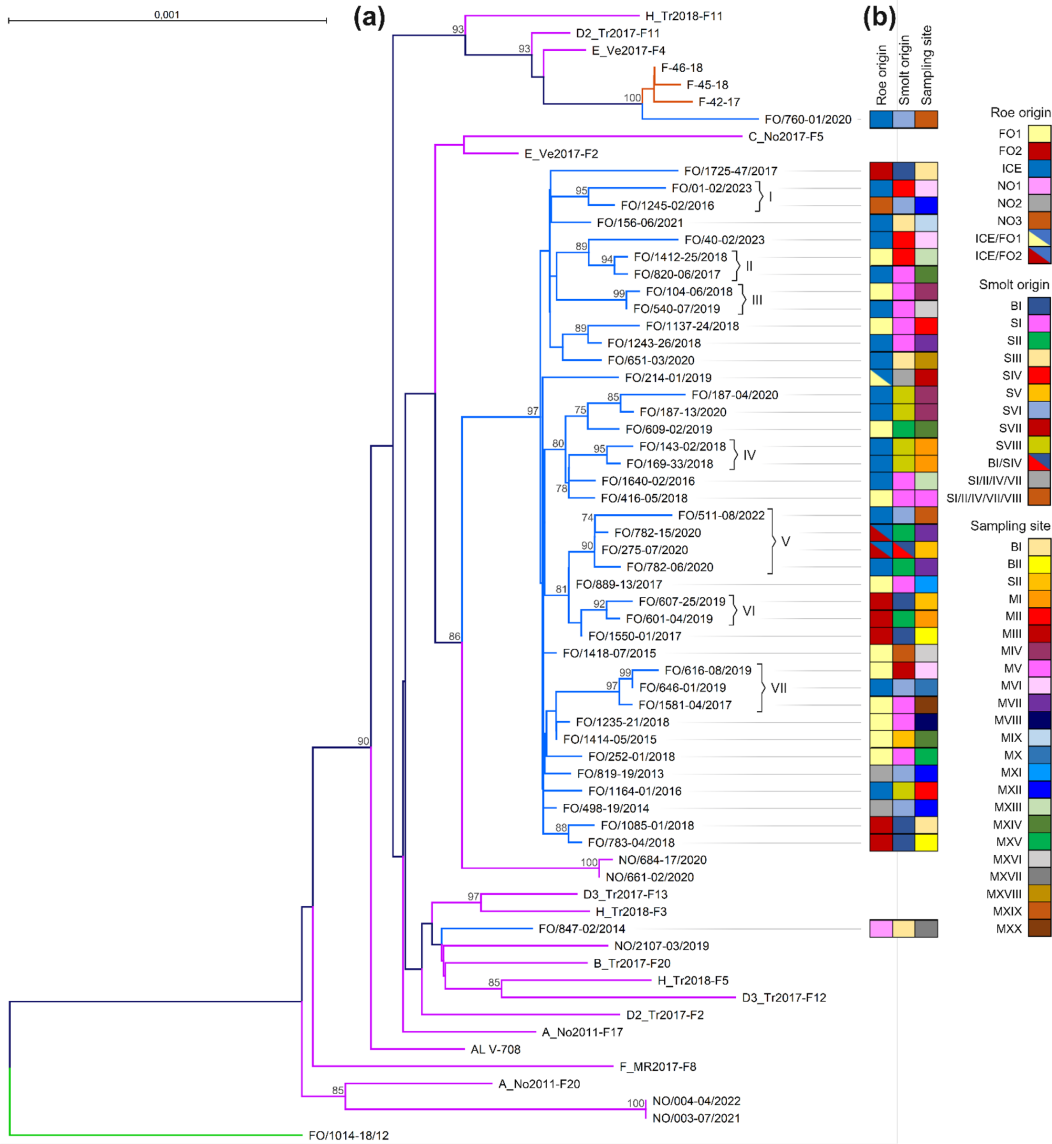
**(a**) Maximum likelihood tree constructed from whole-genome sequences of piscine myocarditis virus (PMCV) from the Faroe Islands (blue branches), Norway (purple branches), Ireland (orange branches) and returning wild Atlantic salmon (green branch). All but two Faroese genomes clustered together and are referred to as the Faroese clade. Seven subclusters with bootstrap values over 90% were identified within the Faroese clade and labelled I–VII. The root is set above FO/1014-18/2012, and the tree is presented as unrooted. Bootstrap values over 70 have been displayed. The scale bar represents 0.001 substitutions per nucleotide site. (**b**) Metadata about the Faroese genomes are depicted with coloured blocks in the categories: roe origin, smolt origin and sampling site. Colour code assignments of categories are described in the legends, and coloured blocks with two different coloured triangles represent samples where both options are possible. FO1 and 2 denote roe origin from the Faroe Islands, ICE from Iceland and NO1, 2 and 3 from Norway.

Phylogenetic analysis of the 48 PMCV genomes presented in this study as well as 14 additional Norwegian and 3 Irish genomes (see above) revealed that all but two of the Faroese genomes formed a monophyletic cluster – the Faroese clade (Fig. 3a).

The bootstrap support for the Faroese clade was high (97%), and it was specifically defined by six unique SNPs spread over the genome, whereof only one resulted in an amino acid substitution: ORF1: T1005C, G1645A (Ala401Thr), C2790T; ORF2: A3305G; UTR2: C5447T; ORF3: C5709T (Table S3, Fig. 3a). Two Faroese genomes, FO/847-02/2014 and FO/609-02/2019, formed outliers and clustered with Norwegian and Irish genomes, respectively (Fig. 3a). The PMCV genomes from the infection challenge in Norway (NO/661-02/2020 and NO/684-17/2020), originating from a field outbreak in 2015 (Table 1), appeared to share the most recent common ancestor with the Faroese clade (Fig. 3a). The PMCV genome from the returning wild salmon (FO/1014-18/2012) differed considerably from the rest of the genomes and formed an outgroup (Fig. 3a).

A pairwise comparison of all genomes in the Faroese clade (Table S4) revealed that no two samples were 100% identical but that the two most divergent genomes (FO/187-04/2020 and FO/40-02/2023) differed by 22 SNPs (99.7% identical nucleotides). In comparison, the two most divergent Norwegian genomes (F_MR2017-F8 and C_NO2017-F5) differed by 59 SNPs (99.1% identical nucleotides). Furthermore, genome-wide analysis detected no insertions and only one deletion in 3′ UTR in genome FO/651-03/2020 (pos. 6559) (Table S3). No apparent intra-individual variants were detected, and in samples representing more than one individual, i.e. the three samples with pooled organs from five individuals and the two water samples representing ten individuals (Table 1), no inter-individual variants were detected. However, two samples (FO/01-02/2023 and FO/40-02/2023) taken a month apart from the same pen in the same production cycle clustered in two distinct subclusters within the Faroese clade, despite having the same roe and smolt origin (Fig. 3a, b).

### No evidence of continuous reintroduction of new persisting PMCV strains in the Faroe Islands

To infer potential introduction and transmission pathways, the Faroese genomes were assigned a colour code reflecting roe origin, smolt origin and sampling site (Fig. 3b). The first detection of PMCV was at a marine farming site (MXII) in 2013 (FO/819-19/2013 and FO/498-19/2014), and the second detection (FO/847-02/2014) was the following year at another marine farming site (MXVII) (Table 1, Fig. 3a). In both cases, the roe was imported from Norway, but from two different producers (NO1 and NO2, respectively) (Fig. 3b). Whereas FO/819-19/2013 and FO/498-19/2014 clustered within the Faroese clade, the first outlier from 2014, FO/847-02/2014, clustered among Norwegian genomes (Fig. 3a, b). The second outlier from 2020, FO/760-01/2020, clustered with Irish genomes despite having Icelandic roe origin (Fig. 3a, b). Of the 42 Faroese PMCV genomes, 30 (71.4%) originated from Faroese FO1 and/or Icelandic roe, while the remaining 12 genomes came from Faroese FO2 (eight genomes) or three different Norwegian producers (four genomes) (Fig. 3b). The fact that over 70% of the Faroese PMCV genomes in farmed salmon originated from brood fish where PMCV has never been detected suggests that these offspring were not infected by their parents but more likely later in the production cycle.

Even though the Faroese clade was highly homogenous, it contained seven subclusters supported by bootstrap values>90% (I–VII, Fig. 3a). By exploring common denominators in the associated sample metadata for each subcluster, no discernible transmission patterns emerged (Fig. 3a, b). Samples in subclusters I, II, V and VII did not share any roe or smolt origin nor sampling sites. Only samples in subcluster VI shared the same roe origin. Samples in subclusters III and IV shared one smolt origin each. Whereas samples in IV were from the same pen in the same production cycle, but at different times, samples in III had two different roe origins as well as two different sampling sites (Table 2). Samples in subcluster III all originated from smolt farm SI, which, of the eight smolt farms included in this study, was highly overrepresented and accounted for the smolt origin of ~26% of the samples. Genetically, however, the genomes were scattered throughout the Faroese clade (Fig. 3a, b).

Overall, the metadata failed to elucidate any explanatory transmission pattern based on the genetic evolution presented by the seven subclusters. It suggests that there is no continuous reintroduction of new PMCV strains to Faroese farmed salmon, but rather that the PMCV strain dominating in Faroese farmed salmon was introduced from Norway some time before 2013 and has spread and evolved locally in the Faroe Islands.

### PCA of genomes in the Faroese clade supports the main findings by the phylogenetic analysis

**Fig. 4.**
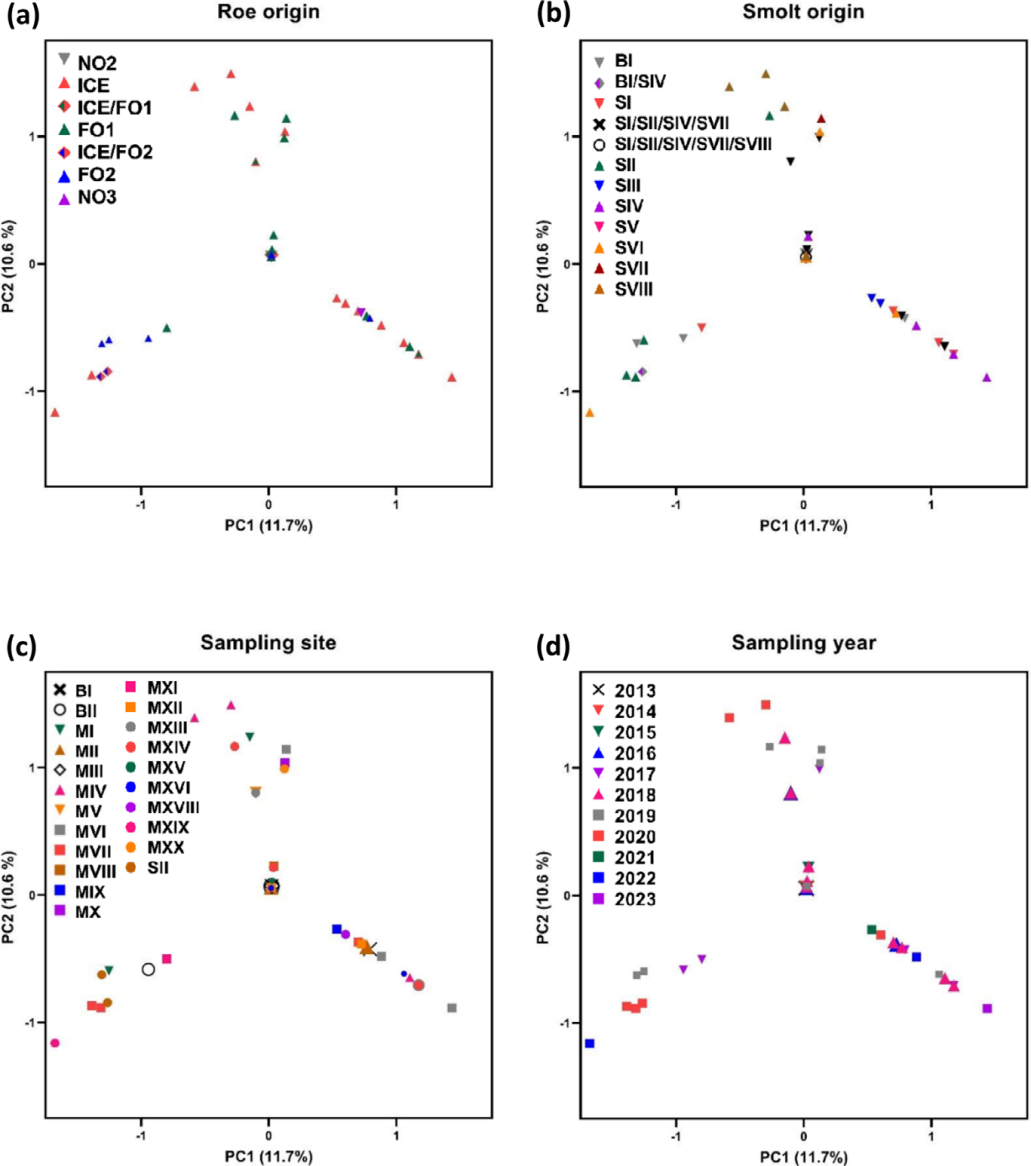
Principal component analyses based on all informative non-outlier SNP loci. All samples belong to the Faroese clade. The percentage of total variation explained by each of the two principal components is indicated. Samples are identified by (a) roe origin, (**b**) smolt origin, (**c**) production site and (d) year sampled. Symbol explanation within each plot.

In agreement with the phylogenetic analysis, PCA confirmed the homogeneity of genotypes in the Faroese clade. PC1 and PC2, the two principal components explaining most of the variation, only accounted for 11.7 and 10.6% of the total variation, respectively (Fig. 4). Further, the main transmission mode for PMCV could not be explained by spatiotemporal distribution of samples nor their origin (roe or smolt), as biplots of PCA showed that no clustering of genotypes was formed when assigning them to these respective categories (Fig. 4).

### No virulence-determining amino acid substitutions in the Faroese clade

At amino acid level, 51 substitutions were identified in the 48 whole-genome sequences when compared to the reference, AL V-708 [3], with variability observed at 50 of the sites within the novel sequences (Table 2). Considering only the 40 genomes belonging to the Faroese clade (Fig. 3a), amino acid substitutions were observed at 28 sites. At each of the 28 sites, only two aminoacids were detected, where the low-frequency amino acid was observed in only one to four of the 40 samples (Table 2). Of the 28 amino acid substitutions, 12 were in ORF1 (861 aa), 7 in ORF2 (726 aa) and 8 in ORF3 (302 aa), thus demonstrating a relatively higher variability for ORF3 compared to ORF1 and ORF2 (Table 2).

**Table 2.** Amino acid substitutions in samples presented in this study relative to the reference sequence, AL V-708. Samples are listed by country and in chronological order. The returning wild salmon sample is highlighted in green, Norwegian samples are highlighted in purple and the two Faroese outliers are highlighted in red. ‘.’ indicates nucleotides identical to AL V-708

­ 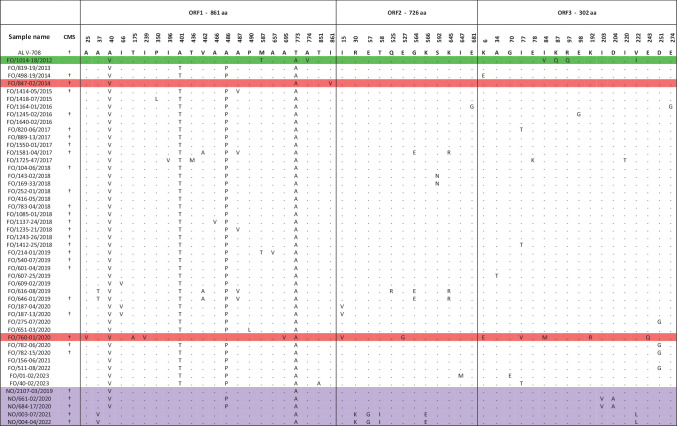

†CMS, as confirmed by gross pathology.

The wild salmon amino acid sequence (FO/1014-18/2012) differed from the reference sequence AL V-708 [3] and all other sequences in the dataset. It exhibited a VQQ motif at positions 84, 87 and 97 in ORF3, as well as an Ala774Val substitution in ORF1 and a Val222Ile substitution in ORF3 (Table 2) . The wild salmon exhibited no clinical signs of CMS. All samples in the Faroese clade had an IKR motif at positions 84, 87 and 97; hence, this motif was observed for samples with and without clinical signs. The outlier from 2020, FO/760-01/2020, with CMS had a MKR motif at positions 84, 87 and 97 in ORF3. Taken together, none of the polymorphic sites could be considered virulence-determining in the sense that one or more amino acids were fixed for all samples with or without clinical signs (Table 2).

### ORF3 exhibited lower evolutionary constraint than ORF1 and 2 in the Faroese clade

The overall genetic variability in the individual ORFs and UTRs is presented in Table 3.

At the nucleotide level, the ORFs exhibited similar sequence variability (~98% conserved). The UTRs were generally more conserved (~99%), except for UTR1, which had the lowest percentage of identical nucleotides (96.4%). At amino acid level, ORF1 and ORF2 were more conserved than their nucleotide sequences (~99%), while ORF3 displayed a higher variability (97% conserved). Furthermore, completely conserved regions were observed in ORF1 (67 to 349 aa) and ORF2 (16 to 524 aa), with no such region in ORF3 (Table 2). This was reflected in the selection pressure *ω*, where dN/dS ratios for ORF1 and ORF2 strongly indicated purifying selection (0.13 and 0.08, respectively). Although all *ω* values <1 indicate purifying selection, ORF3 had a considerably higher *ω* (0.43) (Table 3). Additional *Z*-tests for selection further substantiated that there was no sign of positive selection in any of the ORFs, but whereas ORF1 and ORF2 displayed significant evidence of purifying selection (<0.001), neutral selection could not be rejected for ORF3 (0.18) nor could purifying selection be verified (0.08) (Table 3).

**Table 3.** Genetic variation and selection pressure of the 40 samples in the Faroese clade. Selection pressure *ω* was calculated by SLAC likelihood analysis. *P*-values were generated from *Z*-tests, averaging over all sequence pairs

	5′ UTR*	ORF1	UTR1	ORF2	UTR2	ORF3	3′ UTR*	Total
% identical nucleotides†	98.9	98.1	96.4	97.9	98.8	97.8	99.6	98.1
% identical amino acids†	–	98.5	–	99.0	–	97.0	–	98.5
*ω* (dN/dS)‡	–	0.13	–	0.08	–	0.43	–	–
P neutral§	–	<0.001**	–	<0.001**	–	0.18	–	–
P positive¶	–	1.0	–	1.0	–	1.0	–	–
P purifying¶	–	<0.001**	–	<0.001**	–	0.08	–	–
Selection	–	Purifying	–	Purifying	–	na	–	–

*UTR regions 5′ and 3′ are not complete with coverages of 412nt of 444nt (93%) and 117/179nt of 238nt (49/75%), respectively.

†Percentage of nucleotides (UTRs and ORFs) or amino acids (ORFs) that are identical in all samples.

‡Selection pressure *ω* (dN/dS).

§*P*-values for rejection of null hypothesis (strict neutrality).

¶*P*-values for rejection of null hypothesis (strict neutrality) in favour of an alternative hypothesis (positive or purifying selection).

**P<0.05 are considered significant.

## Discussion

The number of CMS cases in the Faroe Islands has increased markedly over the last decade. To gain a deeper understanding of PMCV epidemiology, we generated 48 novel high-quality PMCV genomes. These genomes were obtained using our in-house developed amplicon-based PCR method for whole-genome sequencing on the Illumina and Oxford Nanopore platforms. Forty-two of the 48 genomes represented a broad spatiotemporal selection of Faroese samples from farmed Atlantic salmon pertaining metadata. The phylogenetic analyses demonstrated that all but two of the Faroese PMCV genomes formed a homogenous monophyletic cluster supported by six unique SNPs suggesting a single introduction of PMCV to the Faroe Islands shortly before the reemergence of CMS in 2013. No apparent link was observed between the clustering of genotypes and the origin of roe or smolt, sampling year or site suggesting that, after the initial introduction of PMCV to the Faroe Islands, local spread between farming sites accounted for the majority of new PMCV infections. Despite two Faroese genomes clustering with Norwegian and Irish genomes, the current dataset showed no apparent evidence of repeating reintroduction of PMCV to the Faroe Islands that could be correlated to the increasing roe import from Norway. Furthermore, no apparent virulence-determining amino acid polymorphisms were identified in the Faroese PMCV genomes as no association was found between specific amino acid substitutions or motifs and the presence or absence of clinical signs. There was significant evidence of purifying selection acting on ORF1 and ORF2 and maintaining genetic conservation in these regions. However, the evidence for purifying selection was less pronounced in ORF3.

One of the Faroese PMCV genomes (FO/1014-18/2012) was from a wild Atlantic salmon caught in 2012 in a Faroese river when returning to spawn. To the best of our knowledge, the current study presents the first whole-genome sequence of PMCV from a wild Atlantic salmon. As the ocean north of the Faroe Islands supports a feeding ground for wild Atlantic salmon populations from the PMCV-positive countries Norway, Ireland, and the UK [34], this specimen could potentially have mixed with PMCV-positive wild Atlantic salmon from other stocks and could inform on the potential role of wild Atlantic salmon as a PMCV vector species. However, whole-genome analysis dismissed PMCV in this sample as an immediate ancestor of PMCV currently circulating in the Faroe Islands, as the sample formed an outgroup to the Faroese, Norwegian and Irish genomes from farmed salmon. Hence, in agreement with Garseth *et al.* [19], these findings do not point to wild Atlantic salmon as an important source of transmitting PMCV to farmed Atlantic salmon.

Our results agree with previous studies that have found PMCV to be genetically homogeneous within both Norway [21] and Ireland [25], though these rely solely on analysis of ORF1 and ORF3. Within Norway, a single genogroup was proposed, though some genetic structure was observed, that coincided partly with the geographical origin of the samples [21]. Within the Irish samples, no such structure was observed, potentially owing to the relatively small Atlantic salmon farming industry [25].

Horizontal transmission of PMCV and subsequent development of CMS has been shown by experimental cohabitation [3] and in field studies [2,9] and has been suggested to be the main route of spreading PMCV [19]. However, the fact that CMS is diagnosed in younger fish [35] with mortalities seen as early as for 100–300 g fish [17] has prompted speculations on potential vertical transmission of the virus. This speculation was further substantiated by the detection of PMCV in brood fish, roe, newly hatched offspring and in individuals up to 40 g [4, 21,36]. However, Ct values were high in roe and offspring, and it is not clear whether the results reflect true infective virus particles causing CMS or RNA template remains [19]. In contrast, another study was not able to find any indications of vertical transmission, as PMCV-RNA was not detected in roe or offspring from brood fish with PMCV-positive heart, milt and ovarian fluid [37]. One major difference between the three studies was the use of disinfection protocols demonstrating the fundamental importance of systematic biosecurity measurements including efficient cleaning and disinfection of fertilized roe to avoid the spread of infectious agents [36,37]. Thus, possible vertical transmission is only of minor importance, corroborated by the fact that PMCV has not been detected in Chile despite the considerable import of Atlantic salmon roe from Norway [17,19]. During the present study period, increasing amounts of roe were imported to the Faroes from Norwegian and Icelandic brood fish, and the reemergence of CMS in 2013 was in salmon from Norwegian roe. Based on the genetic similarity, PMCV may have been introduced to the Faroe Islands from Norway shortly before the first detection in 2013. Similar results have been demonstrated in Ireland where phylogeny suggested that PMCV was introduced from Norway in two waves [25]. Phylogenetic analysis of the Faroese clade together with the metadata suggests a single introduction of PMCV into the Faroes and subsequent horizontal spread between farming sites corroborating previous studies [19,25].

Although our results agree with those of others that PMCV mainly spreads horizontally in the field [9,19], the exact mechanisms are not clear. Yatabe *et al.* [18] pointed to movements of subclinically infected fish as a main driver of transmitting PMCV between sites in Ireland. The only fish movements in the Faroe Islands are the transfer of smolts from the hatchery to the marine grow-out sites and of fish to slaughter either by well boats with mandatory closed valves or in trucks (FFVA, unpublished data). Based on the fact that samples with the same smolt origin show no genetic clustering, it suggests that live fish movements are not a major contributor to the spread of PMCV in the Faroe Islands. Though PMCV was transmitted horizontally in a recent challenge model, it was only to a very low extent [38] compared to reports from the field [9]. Consequently, the authors proposed viral spread through water by yet unknown mechanisms in the field [38]. One significant change in Faroese aquaculture management practices within the last decade is the introduction of large vessels for mechanical delousing. Delousing treatments and traffic between farming sites have increased significantly concurrently with the increase in CMS cases (24; FFVA, unpublished data). Thus, the potential spread of PMCV between farming sites with these vessels, which are problematic to wash and disinfect [39], could be considered a risk factor like the correlation between well boat movement and PD transmission in Norway [40,41] and ISA in Scotland [42].

Totiviruses are believed to have co-evolved with their unicellular hosts like fungi and Protozoa, where infections have been latent and vertical. Whereas totiviruses possess two ORFs, the newly discovered toti-like viruses in, for example, arthropods, worms and fish possess additional coding sequences that are believed to play a role in enhancing infection [20]. Seeing as PMCV and other fish toti-like viruses probably have origins in an arthropod host, it is hypothesized that sea lice could be acting as a vector for PMCV [43]. For PMCV, there is *in vitro* and *in vivo* evidence that transmission is extracellular [3,36], and the extra coding sequence, ORF3, is believed to play some part in this [19]. In this study, the highest amino acid diversity was observed in ORF3 suggesting a potentially different evolutionary trajectory or lower selective constraint on this region than in the other two ORFs. This might be expected, as ORF1 and ORF2 possess conserved vital functions for virus replication. A putative virulence motif has been suggested for the protein encoded by ORF3, where positions 84, 87 and 97 harbour either the IKR or the VQQ amino acid motif [21]. In the current dataset, VQQ was only observed for the returning wild salmon, whereas IKR was the only motif present in the genomes in the Faroese clade. The IKR motif was thus observed for samples with and without CMS and could not be directly related to virulence.

The overall aim of the current study was to provide a comprehensive genetic characterization of the PMCV virus in the Faroe Islands, focusing on its reemergence in 2013 and subsequent spread. Accordingly, the sample selection strategy prioritized obtaining a broad spatiotemporal coverage rather than analysing multiple samples from individual sites. This may potentially have skewed estimates of genetic variability towards homogeneity, as others have found that within-site variation can be high [21,25, 28]. Any underestimation of genetic variability is more likely from 2019 onwards where only a subset of samples was included. However, the potential underestimation is only minor, as three pooled samples representing five individuals each as well as two seawater samples representing ten individuals each did not disclose any genetic variability among individuals. Interestingly, we show here that it is feasible to monitor and detect PMCV in environmental water samples, as demonstrated with the two positive seawater samples with relatively low Ct values, though previous attempts to detect PMCV in environmental samples such as sediment and biofilm have not been successful [19]. This is promising for future transmission studies as well as for non-lethal monitoring purposes. The two water samples included here were part of regular PMCV monitoring of anaesthetic tank water used for sea louse counts, used by farmers as an early warning method to make informed decisions on, for example, the order of net pens to be treated in delousing events.

In conclusion, the established amplicon-based method proved to be fast and well-suited for whole-genome sequencing of PMCV from a wide range of sample types including clinical and subclinical salmon as well as environmental water samples. The phylogeny and metadata showed no continuous reintroduction of persisting PMCV strains to Faroese farmed salmon and no evidence of vertical transmission being the main transmission mode. No virulence-determining amino acid polymorphisms were identified in the Faroese PMCV genomes as we found no apparent association between specific amino acid motifs and disease outcome. PMCV in the Faroe Islands forms a monophyletic cluster with minor genetic variability that could not be explained by the investigated parameters of smolt or roe origin, sampling site or year. As our results indicate that PMCV mainly spread locally between marine farming sites in the Faroes and the fact that fish are not moved between farming sites, future research should explore the movement of contaminated vessels as a potential transmission vector for spreading PMCV in Faroese aquaculture. Nevertheless, the fact that PMCV may have been introduced to the Faroes via imported Norwegian roe and subsequently spread fast between farming sites underlines the importance of proper biosecurity measurements at all levels in Atlantic salmon production to mitigate the spread of PMCV and other infectious pathogens.

## supplementary material

10.1099/jgv.0.002068 Uncited Supplementary Material 1.

## Abbreviations

CMS: cardiomyopathy syndrome
Ct: cycle threshold
eRNA/eDNA: environmental RNA/DNA
FFVA: Faroese Food and veterinary authority
IKR: Isoleucine-Lycine-Arginine
ISA: infectious salmon anaemia
ISAV: Infectious Salmon Anaemia Virus
NGS: next-generation sequencing
PC: principal components
PCA: principal component analysis
PD: pancreas disease
PMCV: piscine myocarditis virus
RLT: RNA Lysis buffer
RT-qPCR: real-time reverse transcription quantitative PCR
SLAC: single likelihood ancestor counting
VQQ: Valine-Glutamine-Glutamine
